# Recent court ruling could increase the size and administrative complexity of the 340B program

**DOI:** 10.1093/haschl/qxae157

**Published:** 2024-12-03

**Authors:** Sayeh Nikpay, John P Bruno, Colleen Carey

**Affiliations:** Division of Health Policy and Management, University of Minnesota School of Public Health, Minneapolis, MN 55455, United States; Cornerstone Research, Chicago, IL 60602, United States; Department of Economics and Brooks School of Public Policy, Cornell University, Ithaca, NY 14853, United States

**Keywords:** 340B program, Medicare Part D, prescription drugs

## Abstract

The 340B program allows certain hospitals and clinics to use outpatient drugs purchased at substantial discounts on insured patients, generating profits to fund care. The size of these profits depends on the number of prescriptions filled by participating hospital or clinics’ insured patients that also meet the Health Resources and Services Agency's definition of an eligible patient. A recent court case has challenged the Agency's longstanding definition of a patient, resulting in new definition that could significantly expand the size of the program and create conflicts when an insured patient satisfies the new definition for more than one hospital or clinic participating in the program. We use Medicare Part D data from 2018 to simulate the proportion of prescription drug fills eligible for 340B discounts and total program spending under both existing and new definitions. We found that the new definition could increase the share of 340B-eligible fills in Medicare Part D by 25%, from 12% of fills to 16%, and that the share of fills subject to a conflict could double, from 1% of fills to 1%-2%. Our results suggest that the new definition could increase covered entities’ 340B profits by roughly a third.

## Introduction and background

The 340B program entitles certain clinics and hospitals, called “covered entities” (CEs), to purchase outpatient drugs at substantial discounts to defray the costs of providing uncompensated care. Covered entities can also generate profits when they bill insurers for these drugs. Although data are not currently publicly available, figures published by the Health Resources and Services Administration (HRSA) suggest that 340B profits could be as high as $50 billion annually if drugs purchased through the program are reimbursed at list prices.^1^

340B profits depend directly on the number of a CE's insured patients that meet HRSA's discount eligibility definition. Although the statute enabling 340B is silent on who counts as a patient, HRSA provided a definition in guidance. A core requirement is that the patient receives care from a provider that is affiliated (through employment, a contract, or a referral relationship) with the CE.^2^ Health Resources and Services Administration uses audits to ensure that CEs comply with this definition.

A recent court case has raised the question of what it means to “receive care” from an affiliated provider. After an HRSA audit in 2018, Genesis Healthcare, a Federally Qualified Health Center, lost 340B eligibility for violating HRSA's definition. Suing HRSA, Genesis argued that HRSA applied additional audit criteria beyond HRSA's definition. Indeed, the suit revealed that HRSA also imposed the requirement that the CE's affiliated provider wrote the prescription. Siding with Genesis Healthcare in 2023, the Fourth Circuit Court of Appeals determined that the additional requirement was not lawful and that the definition of “receiving care” in the 340B program should hew more closely to the plain language definition: “awaiting or under medical care and treatment.”^3^

Although the revised definition under the ruling applies only to Genesis Healthcare, the definition could increase the size of the 340B program significantly if applied broadly. Under the new definition, the number of 340B-discounted drugs that CEs can use on patients should increase from only those prescribed by affiliated providers to any prescription used on patients receiving care from the CE. Estimates from the gray literature suggest the share of branded drugs eligible for 340B discounts could double^4^ or even triple.^5^

In addition, the revised definition should increase the administrative complexity of the program by increasing “conflicted” prescription fills. For these fills, the prescription meets the revised definition for 2 or more CEs. For example, if a patient receives care from 2 different CEs, each would be equally entitled to use discounted drugs on the patient, regardless of which CE's affiliated provider wrote the prescription.

In this Brief Report, we modeled the impact of pre- and post-*Genesis* definitions on 340B-eligible fills and spending in Medicare Part D.

## Data and methods

We identified Part D fills from 2018 for patients enrolled in fee-for-service Medicare. To assess whether each fill was eligible for 340B discounts under the pre- or post-*Genesis* definitions, we needed to assess (1) whether the prescriber was affiliated with a CE, (2) whether the pharmacy at which the prescription was filled was registered with HRSA as eligible to dispense 340B-discounted prescriptions, and finally (3) whether the patient had received care from the CE. To identify possible affiliations, we found all the CEs where the prescriber supplied outpatient services (see Supplementary material).^6^ To identify pharmacies eligible to dispense 340B-eligible prescriptions for each CE, we extracted all in-house pharmacies and community pharmacies with a contract to dispense 340B-eligible prescriptions on behalf of the CE, called “contract pharmacies,” registered under the CE in HRSA's Outpatient Pharmacy Administration System. We identified patients receiving care from CEs by identifying inpatient and outpatient claims with the CE over 2 lookback periods: the same quarter of the fill (a restrictive estimate) and the past 10 years (an expansive estimate).

Due to the change in the definition of “receiving care,” the pre- and post-*Genesis* definitions differed in the set of Part D fills eligible for 340B discounts. Pre-*Genesis*, a fill was eligible for 340B discounts when the prescriber was affiliated with a CE and the pharmacy was eligible to dispense 340B prescriptions for the same CE. The post-*Genesis* definition included all fills eligible for discounts under the pre-*Genesis* definition but added to it all fills through the CE's in-house or contract pharmacies for patients who had received inpatient or outpatient services at the same CE. We hypothesized the post-*Genesis* definitions are likely to increase “conflicts.” For example, if a patient received services from 2 CEs and filled a prescription at a pharmacy contracted with both CEs, both CEs could claim a right to 340B discounts for the fill.

Our analytic dataset represented 51% of all Medicare Part D fills in 2018. We presented the proportion of 2018 Part D fills for which CEs could use 340B-discounted drugs by pre- and post-*Genesis* definitions and by whether the prescription fill was subject to a conflict.

## Results

Under the pre-*Genesis* definition, 12% of fills were 340B-eligible, with 1% of total fills conflicted (Table 1). Under the post-*Genesis* definition, the 340B-eligible proportion of fills increased from 13.5% with no lookback to 16% with a 10-year lookback. The conflicted proportion of total fills ranged from 1.7% with no lookback to 2.6% with a 10-year lookback.

**Table 1.** 340B-Eligible Medicare Part D fills and spending by definition, and number of covered entities entitled to fill patient prescription with 340B-discounted drugs, 2018.

	340B-Eligible share of Medicare Part D fills (%)		340B-Eligible Medicare Part D spending ($billions)	
	1 CE	>1 CE	1 CE	>1 CE
Pre-*Genesis*	10.4	1.2	18.6	2.1
Post-*Genesis*				
No lookback	11.6	1.4	20.7	2.5
10-Year lookback	13.4	2.4	23.8	4.2

Source: 2018 20% Medicare Prescription Drug Event File and 2008-2018 Inpatient and Outpatient Claims, 340B Outpatient Pharmacy Affairs Information System, 2018 National Council for Prescription Drug Plans Data Q database.

The table presents the proportion of Medicare Part D prescriptions by the number of CEs that were entitled to use 340B drugs to fill the prescription through their in-house or contract pharmacy: 1 or 2 or more. The pre-*Genesis* definition counts as 340B-discounted eligible all filled prescriptions that are prescribed by a 340B-affiliated prescriber and filled at a pharmacy that is either owned or has a contract with the CE. The post-*Genesis* definition counts as eligible all prescriptions attributable under the pre-*Genesis* definition and adds CEs whose affiliated prescribers supplied inpatient or outpatient services to the patient when the prescription for that patient is filled at the same CE's contract pharmacy. The post-*Genesis* definitions look back over 2 time periods: the current quarter only or the past 10 years.

Abbreviation: CE, covered entity

Measured in terms of spending, total Medicare Part D expenditure on 340B-eligible fills was $22 billion (B) under the pre-*Genesis* definition, including $2B in spending on conflicted fills. Under the post-*Genesis* definitions, the total expenditure on 340B-eligible Part D fills ranged from $24B with no lookback period to $30B with a 10-year lookback period. The total expenditure for conflicted Part D fills ranged from $2B with no lookback period to $4B with a 10-year lookback period.

## Discussion and conclusion

Our results suggest that expanded definitions in the *Genesis* ruling could increase both the size and administrative complexity of the 340B program. Measured as a proportion of total Medicare Part D fills in 2018, the new definition could increase 340B-eligible fills by 25%. This change would represent an $8B increase in Part D spending for which CEs could use drugs purchased through 340B to generate profits. Assuming that 340B discounts in Medicare Part D are comparable to those for physician administered drugs in Medicare Part B (53%),^5,7^ we would expect the new definition to increase CEs’ 340B current $12B profits from Medicare Part D to $16B, or a 33% increase.

We also found that the post-*Genesis* definition could approximately triple the share of Medicare Part D fills that are conflicted. It is unclear how such conflicts will be resolved as multiple CEs attempt to use their own supply of 340B-discounted drugs for this estimated 3% of Medicare Part D fills.

Our results relied on administrative data and should therefore be interpreted with caution. However, our estimate of the pre-*Genesis* share of fills eligible for 340B in Medicare Part D is similar to other published studies.^8^ Another limitation is that we used outpatient services for hospital-provider affiliations, which could under-estimate the share of fills eligible for 340B discounts if many outpatient prescriptions originate from providers who only work in the inpatient setting. In addition, our analysis only considered hospital CEs, which accounted for ∼80% of 340B-eligible drug purchases in 2021.^1^ Finally, our analysis used data from 2018 rather than the most recent year of claims data available. However, 2018 does not reflect COVID-19 disruptions or certain restrictions on the use of 340B-discounted drugs through contract pharmacies imposed by pharmaceutical manufacturers.

Placed in the context of growing scrutiny over 340B profits, increasing the size and complexity of the 340B program raises the need for clarity on the definition of an eligible patient as well as the program's purpose.

## Supplementary Material

qxae157_Supplementary_Data
