# Supplementary material for: Recent court ruling could increase the size and administrative complexity of the 340B program
Source: Health Aff Sch. 2024 Dec 3;2(12):qxae157. doi: 10.1093/haschl/qxae157 (PMC11642606; doi:10.1093/haschl/qxae157)
Supplement: qxae157_Supplementary_Data [file qxae157_supplementary_data.zip › coi_disclosure_Nikpay.pdf]

ICMJE DISCLOSURE FORM

Date: 9/29/2024

Your Name: Sayeh Nikpay

Manuscript Title: Recent Court Ruling Could Increase the Size and Administrative Complexity of the 340B Program

Manuscript Number (if known): Unknown

In the interest of transparency, we ask you to disclose all relationships/activities/interests listed below that are related to the content of your manuscript. "Related" means any relation with for-profit or not-for-profit third parties whose interests may be affected by the content of the manuscript. Disclosure represents a commitment to transparency and does not necessarily indicate a bias. If you are in doubt about whether to list a relationship/activity/interest, it is preferable that you do so.

The author's relationships/activities/interests should be defined broadly. For example, if your manuscript pertains to the epidemiology of hypertension, you should declare all relationships with manufacturers of antihypertensive medication, even if that medication is not mentioned in the manuscript.

In item #1 below, report all support for the work reported in this manuscript without time limit. For all other items, the time frame for disclosure is the past 36 months.

		**Name all entities with whom you have this relationship or indicate none (add rows as needed)**	**Specifications/Comments (e.g., if payments were made to you or to your institution)**
**Time frame: Since the initial planning of the work**			
**1**	All support for the present manuscript (e.g., funding, provision of study materials, medical writing, article processing charges, etc.) **No time limit for this item.**	☒ **None**	
			Click the tab key to add additional rows.
**Time frame: past 36 months**			
**2**	Grants or contracts from any entity (if not indicated in item #1 above).	☐ **None**	
		Arnold Ventures (PI)	I have several active grants from Arnold Ventures to study issues related to the 340B program on pharmacies, safety-net providers, and Medicaid spending (ACTIVE)
		Minnesota Department of Health, Health Economics Program (PI)	I am contracted with the Department of Health's Economics Program to process and analyze data for Minnesota's 340B Covered Entity Report. (ACTIVE)
		National Heart Lung and Blood Institute (PI, Co-I)	R01 to quantify the structure of STEMI interhospital transfer networks and estimate the impact of hospital closures on interhospital transfer decisions. (ACTIVE)

		Name all entities with whom you have this relationship or indicate none (add rows as needed)	Specifications/Comments (e.g., if payments were made to you or to your institution)
			R21 to estimate the impact of insurance on interfacility transfer decisions. (CONCLUDED)
		Centers for Medicare and Medicaid Services (Co-I)	Contract to provide technical assistance to users of the SEER-Medicare and SEER-Medicaid. (ACTIVE)
		Agency for Healthcare Research and Quality (Co-I)	R01 to quantify insurance networks. (CONCLUDED)
		National Institute for Health Care Management (Co-I)	Grant to estimate the impact of Ransomeware attacks on hospital finances and patient outcomes. (CONCLUDED)
**3**	Royalties or licenses	☒ **None**	
**4**	Consulting fees	☐ **None**	
		Health Affairs Scholar	I receive an annual payment for serving as an Associate Editor for Health Affairs Scholar, a peer-reviewed journal.
**5**	Payment or honoraria for lectures, presentations, speakers bureaus, manuscript writing or educational events	☒ **None**	
**6**	Payment for expert testimony	☒ **None**	
**7**	Support for attending meetings and/or travel	☒ **None**	

		Name all entities with whom you have this relationship or indicate none (add rows as needed)	Specifications/Comments (e.g., if payments were made to you or to your institution)
**8**	Patents planned, issued or pending	☒ **None**	
**9**	Participation on a Data Safety Monitoring Board or Advisory Board	☒ **None**	
**10**	Leadership or fiduciary role in other board, society, committee or advocacy group, paid or unpaid	☐ **None**	
		Minnesota Prescription Drug Affordability Board	I am the Vice Chair of Minnesota's Prescription Drug Affordability Board.
**11**	Stock or stock options	☒ **None**	
**12**	Receipt of equipment, materials, drugs, medical writing, gifts or other services	☒ **None**	
**13**	Other financial or non-financial interests	☒ **None**	

Please place an "X" next to the following statement to indicate your agreement:

☒ I certify that I have answered every question and have not altered the wording of any of the questions on this form.
