# Supplementary material for: Recent court ruling could increase the size and administrative complexity of the 340B program
Source: Health Aff Sch. 2024 Dec 3;2(12):qxae157. doi: 10.1093/haschl/qxae157 (PMC11642606; doi:10.1093/haschl/qxae157)
Supplement: qxae157_Supplementary_Data [file qxae157_supplementary_data.zip › Patient Attibution and 340B Supplemental Appendix R1.docx]

**Supplemental Appendix**

Data Linkages

Our analytic dataset was created by linking several datasets. The first dataset was a 20% sample of 2018 Medicare Prescription Drug Event (PDE) and 2008-2018 fee-for-service (FFS) Medicare outpatient and inpatient claims. We limited our sample to prescriptions for 2018 Medicare fee-for-service beneficiaries with 12 months of enrollment in Parts A, B, and D. These prescriptions, numbering 154,915,952, represented 51% of all Medicare Part D claims.

The second dataset was derived from the 340B Outpatient Prescription Drug Administration System, which lists the initiation and termination dates, in-house pharmacies, and contract pharmacies registered under CEs. We used this dataset to create a hospital-quarter-year file with the Medicare Provider Number (MPN) of all CEs appearing in the claims data (2,174 unique MPNs). We also create a pharmacy-quarter-year file with the 340B registration status for all in-house and community pharmacies listed in the National Council for Prescription Drug Plans (NCPDP) database – a listing of all pharmacies in the United States. Linking to NCPDP was necessary as the 340B data do not contain pharmacy identifiers necessary to link pharmacies to the PDE data.

The OPAIS data was matched to the NCPDP data by an “augmented location matching” approach. First, the address fields in both datasets were cleaned and then used to add geocode (latitude and longitude) information. We then calculate the pair-wise straight-line distance between all pharmacies in the same state in each dataset, and filter to pairs that are within 0.2 miles of each other. This is important, as small differences in how addresses appear in different datasets (e.g. if one address includes a suite number) can lead to differences in geocodes. We then implement fuzzy-matching (using jaro-winkler, levenstein, and cosine string distance) on both pharmacy name and address to identify pairs of pharmacies that are matches. When a pharmacy in OPAIS is matched to multiple NCPDP pharmacies, manual review is used to identify which pair is correct.

In 2018, we identified 18,624 unique in-house or contract pharmacies who are contracting with, on average, 1.75 CEs.

Method for Assigning 340B Eligibility under Both Definitions.

In the sections below we describe how these datasets are linked to construct the pre- and post-*Genesis* prescription eligibility definitions. Appendix Figure 1, shows a schematic representation of the criteria necessary to satisfy both definitions:

*Pre-Genesis Definition*

We define a prescription as eligible for 340B discounts under the pre-*Genesis* definition if its prescriber is affiliated with a CE and the pharmacy where the prescription is filled has a contract with the same CE.

We began by affiliating prescribers in the 2018 PDE, identified by their National Provider Identifier (NPI), to CEs, identified by MPN. To do this, we gathered all the MPNs where the prescriber had billed in the 2018 Outpatient claims. This method is qualitatively similar to commonly used hospital-physician integration algorithms, except that we do not limit to a minimum billing threshold in order to capture as many affiliations as possible.^1^ This linkage allowed us to determine which CE or CEs could claim each prescription under the pre-*Genesis* prescriber affiliation requirement. Among our sampled prescriptions, approximately half of the prescribers (513,944) are affiliated with one or more CEs (an average of 2.4).

**Appendix Figure 1: Pre- and Post-Genesis Definition Schematic**


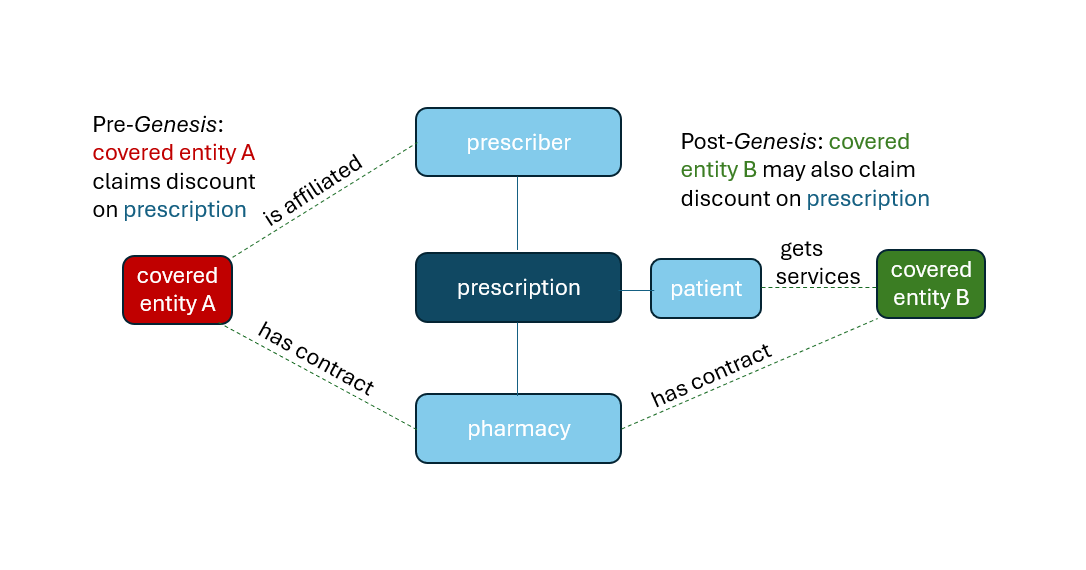


To implement the second element of the discount eligibility definition, we verify that the prescription was filled at a qualifying contract pharmacy. Specifically, we examine whether the pharmacy has a contract with the CE (or CEs) with which the prescriber is affiliated.

*Post-Genesis Definition*

To construct the post-*Genesis* definition, we add another pathway for discount eligibility to the pre-*Genesis* definition. The new pathway is that a prescription can be eligible for discounts if the patient has obtained services from a CE and is filled at a pharmacy that contracts with that CE. To identify these additional CEs, we collected all observed MPNs providing care to the patient over different “lookback” periods: same quarter as the prescription, 3 years, and 10 years.

Under the pre-*Genesis* definition, a prescription is eligible for discounts at two or more CEs if the pharmacy holds contracts with multiple CEs and the prescriber is affiliated with those same CEs. Post-*Genesis*, there are more pathways that result in a prescription being eligible for discounts at multiple CEs. For example, the patient may have obtained services from multiple CEs over the lookback period; alternatively, the prescriber may be affiliated with one CE, but the patient has obtained services from a different CE.

References

1. Neprash HT, Chernew ME, McWilliams JM. Little Evidence Exists To Support The Expectation That Providers Would Consolidate To Enter New Payment Models. *Health Aff (Millwood)*. 2017;36(2):346-354. doi:10.1377/hlthaff.2016.0840
